# Metadata-Private Resource Allocation in Edge Computing Withstands Semi-Malicious Edge Nodes

**DOI:** 10.3390/s24102989

**Published:** 2024-05-08

**Authors:** Zihou Zhang, Jiangtao Li, Yufeng Li, Yuanhang He

**Affiliations:** 1 School of Computer Engineering and Science, Shanghai University, Shanghai 200444, China; zzh@shu.edu.cn (Z.Z.); lijiangtao@shu.edu.cn (J.L.); liyufeng_shu@shu.edu.cn (Y.L.); 2 Science and Technology on Communication Security Laboratory, Chengdu 610041, China; 3 No. 30 Research Institute of China Electronics Technology Group Corporation, Chengdu 610041, China

**Keywords:** Internet of Things, edge computing, privacy-preserving, resource allocation

## Abstract

Edge computing provides higher computational power and lower transmission latency by offloading tasks to nearby edge nodes with available computational resources to meet the requirements of time-sensitive tasks and computationally complex tasks. Resource allocation schemes are essential to this process. To allocate resources effectively, it is necessary to attach metadata to a task to indicate what kind of resources are needed and how many computation resources are required. However, these metadata are sensitive and can be exposed to eavesdroppers, which can lead to privacy breaches. In addition, edge nodes are vulnerable to corruption because of their limited cybersecurity defenses. Attackers can easily obtain end-device privacy through unprotected metadata or corrupted edge nodes. To address this problem, we propose a metadata privacy resource allocation scheme that uses searchable encryption to protect metadata privacy and zero-knowledge proofs to resist semi-malicious edge nodes. We have formally proven that our proposed scheme satisfies the required security concepts and experimentally demonstrated the effectiveness of the scheme.

## 1. Introduction

Edge computing offers significantly enhanced computing power by outsourcing end-device tasks to nearby edge nodes or devices with additional computing resources. The reduced transmission latency in edge-based solutions, as compared with traditional cloud-based alternatives, underscores its efficiency. As intelligent requirements for terminal devices continue to evolve, the limitations of traditional cloud computing become evident, especially in handling a multitude of delay-sensitive and computationally intensive tasks. This shift has given rise to the emergence of edge computing [1]. The application scope of edge computing extends widely across industry and manufacturing, agriculture, healthcare, urban management, and environmental protection [2,3,4,5], leveraging the transformative potential of the Internet of Things (IoT).

The optimization of task offloading and resource allocation is the core problem of edge computing [6], and the scheme based on a smart gateway [7] is considered a promising solution. Specifically, the end device will send the task to the smart gateway, which will carry out measures such as refining and filtering the data according to the application requirements at the smart gateway end, and finally assign the task to the appropriate edge node for processing, in which the gateway should take into account various factors, such as the type of the task, the time sensitivity, and the computational complexity. In order to realize such a system, metadata defining the factors required for the task need to accompany the task data when they are sent [8], and these metadata can help the gateway to make decisions when allocating resources [9]; e.g., time sensitivity points out that the task needs to be processed in real-time, and computational complexity points out that the task needs more computational resources.

The metadata of edge computing contain sensitive information, such as when a user publishes a task, what kind of resource they request, and how much computation resource they need. Unprotected metadata will expose the end device’s private information; e.g., a malicious adversary can launch a side-channel attack by observing how the gateway performs the resource allocation to obtain the user’s privacy information [10]. Some research has shown that it is possible to get a detailed and private picture of a person’s contacts and interests just by analyzing metadata, and it is technically much more accessible to search massive amounts of metadata than to listen in on millions of phone calls [11]. In recent years, some researchers have focused on the metadata privacy in secure messaging [12,13], however, in the area of edge computing, we still face the challenge of lower computation resources on the end devices and lower bandwidth requirements.

A possible method to achieve metadata privacy in edge computing is for the end device to encrypt the metadata using the public key of the gateway and send it to the gateway, which decrypts it and then completes the resource allocation. Although this scheme can resist malicious eavesdroppers, the gateway can still obtain information about the metadata, and once the gateway is corrupted, the attacker can still obtain the user’s privacy. To solve the above problem, a scheme based on searchable encryption has been proposed [14]; by using searchable encryption, the smart gateway can check whether the received encrypted metadata contains a specific keyword without learning any metadata-related content.

It is challenging to run a strong cyber defense system because of the relatively limited computing power of the edge nodes. In addition, the security setup of the edge nodes is also more prone to errors than the cloud [15]. As a result, edge nodes are more easily corrupted and become semi-malicious edge nodes. A semi-malicious edge node is defined as an edge node that will maintain system consistency but will behave maliciously to weaken the privacy of the system. However, the above scheme [14] lacks the consideration of semi-malicious edge nodes, which may allow the gateway to perform equality test searches for specific keywords if one or more edge nodes are corrupted in the group generation phase.

### 1.1. Our Contribution

Existing resource allocation schemes for metadata privacy protection often lack the consideration of malicious smart gateways and semi-malicious edge nodes. In order to address these challenges, we propose a resource allocation scheme for metadata privacy protection based on searchable encryption. This scheme is valuable in edge computing environments with limited computational resources and bandwidth, such as the Internet of Vehicles. The contributions of this work are as follows:

First, edge nodes can generate a group encryption public key by interacting with each other, and subsequently, for a set of selected keywords, each edge node can compute a partial trapdoor, and the smart gateway can aggregate these partial trapdoors to generate a complete trapdoor. End devices need to encrypt the corresponding metadata using the group public key when sending data and send the encrypted metadata to the gateway together with the task data. The gateway uses a complete trapdoor to determine whether the encrypted metadata contains the specific keyword and finally allocates resources according to the predetermined policy. In the process, the gateway does not learn any keyword-related content. Moreover, our proposed scheme achieves constant message expansion, i.e., the ciphertext length of the encrypted keyword does not increase with the increase in edge nodes.

Second, we use extractable zero-knowledge proofs to resist corrupted edge nodes. Specifically, in the group generation phase, each edge node must provide the corresponding proofs of the selected random parameter, and the parameter without the corresponding proofs will not be added to the corresponding computation of the group public key, which prevents semi-malicious edge nodes from enabling the gateway to perform an equality test search on a specific keyword by generating a malicious parameter in the group generation phase.

Finally, we identify the security concepts of a metadata-privacy resource allocation scheme and perform the corresponding security analysis. Our scheme captures important security properties such as user privacy, full key compromise resistance, and semi-malicious edge node resistance. In addition, we give a formal proof of how our proposed scheme satisfies the defined security concepts. In addition, we experimentally evaluate the performance of the proposed scheme.

### 1.2. Related Work

The allocation of limited resources is the most critical component of an edge computing system, and its efficiency directly affects the whole system’s performance. Recently, many resource allocation strategies and algorithms have been proposed to realize resource allocation and management in edge computing [16,17,18]. The use of smart gateways [7] is considered a promising solution for edge computing resource allocation, where the smart gateway can utilize system resources better by deciding the time and type of data to be sent based on application feedback. However, it is worth noting that user privacy is not considered in Aazam et al.’s scheme [7].

Lu et al. [19] proposed a lightweight privacy-preserving data aggregation scheme based on homomorphic encryption and the Chinese Remainder Theorem in response to the limitation that existing schemes only support homogeneous devices and experimentally proved the scheme’s efficiency. However, the scheme does not consider identity privacy and traceability. In order to reduce privacy leakage in edge computing, Lyu et al. [20] proposed a new privacy-preserving data aggregation scheme based on homomorphic encryption and differential privacy, which guarantees the obliviousness of the aggregator. However, the scheme does not consider the edge node that may steal the user’s location privacy. Zhang et al. [21] proposed a resource allocation scheme that can resist malicious edge nodes using blockchain and trust computing. However, since the scheme uses symmetric searchable encryption, it cannot generate complete trapdoors cooperatively. Thus, the scheme is difficult to implement to protect metadata privacy. Kong et al. [22] designed a task offloading strategy based on a multifeedback trust mechanism, which can identify malicious nodes using trust assessment and improve system operation efficiency using the clustering algorithm. Zhou et al. [23] proposed a lightweight, verifiable privacy-preserving outsourcing matching pattern protocol that prevents collusion between the cloud and malicious receivers/senders. Li et al. [24] proposed a verifiable edge outsourcing computing scheme based on blockchain, which protects the privacy of the results. Wang et al. [25] designed a privacy-preserving computational offloading framework based on vehicular fog computing and smart contracts and experimentally demonstrated the effectiveness of the framework. However, we note that all the above schemes lack consideration of metadata privacy issues.

In order to protect the privacy of metadata in edge computing, Zhang et al. [14] proposed a scheme based on searchable encryption that achieves constant message expansion and full key compromise resistance. Angel et al. [26] proposed a new resource allocation scheme that allocates resources to a group of clients and does not disclose to the clients whether there are other clients receiving the resources. Beams et al. [10] proposed a privacy-preserving packet scheduling scheme that prevents a malicious adversary from obtaining the victim’s private information by launching a side-channel attack and observing how the switch schedules packets. Ahmad et al. [27] proposed a new voice communication scheme that enables metadata privacy-preserving voice communication over a completely untrusted infrastructure. However, all the above privacy-preserving resource allocation schemes do not consider malicious edge nodes.

Barman et al. [12] proposed a metadata privacy messaging system based on differential privacy and Private Information Retrieval (PIR) [28] that can support asynchronous messaging chats and can run on untrusted servers. Tests of a prototype system show the effectiveness of the scheme. Jiang et al. [13] implemented a scalable metadata-private messaging system based on hardware enclaves that achieves resistance to malicious clients. Experiments show that the scheme has a lower extra cost and higher efficiency. Cai et al. [29] designed a metadata-hiding data analytic system called Vizard using the distributed point function, which allows data owners to share their data in a privacy-preserving manner, and the experimental results proved the practicality of the scheme. Langowski et al. [30] designed a metadata-private anonymous broadcasting scheme called Trellis based on boomerang encryption and anonymous routing tokens that hides all network-level metadata and supports horizontal scalability. However, these solutions are designed for large network systems and are difficult to apply directly to edge systems with limited computational and bandwidth resources.

## 2. Background

### 2.1. System Architecture

**Figure 1:**
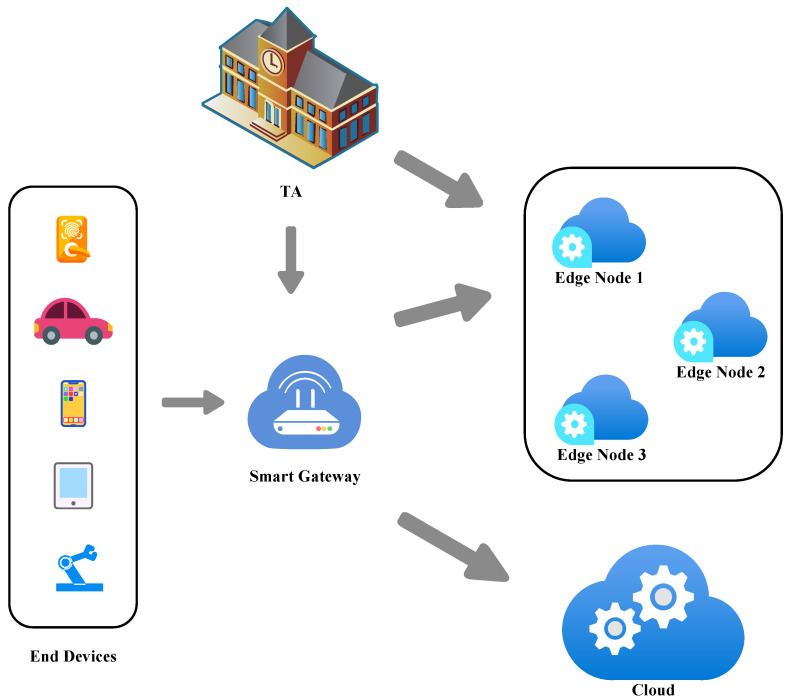
System architecture.

The edge computing system used in our proposed scheme comprises five types of entities: trusted authority (TA), end device, gateway, edge node, and cloud, as shown in Figure 1.

- TA provides registration services for edge nodes and generates system parameters. In our scheme, TA is considered to be entirely trustworthy.
- End devices with limited computing and storage capabilities are users of the edge computing system and need to accomplish various tasks with the help of edge nodes.
- The gateway does the forwarding process for the received task and finds the appropriate edge nodes or cloud. Note that since the resource allocation is done by the gateway and not by the end device itself, only one group exists at every moment. In our scheme, the gateway is considered to be honest but curious [31], meaning that the gateway will honestly follow the protocol to complete the forwarding job but will try to violate the privacy of the end device.
- Edge nodes have a relatively limited computational and storage capacity [32,33], and thus generally need to collaborate to handle tasks. In our scheme, edge nodes may be compromised to become semi-malicious edge nodes. A semi-malicious edge node is defined as an edge node that will maintain system consistency but will behave maliciously (we call it an elimination attack) to weaken the privacy of the system. The elimination attack is defined as follows: Suppose an edge node with identity $ID_{i}$ is corrupted by the smart gateway in the group setup phase. The elimination attack may “eliminate the necessity authorization“ via edge node $ID_{j}$. In other words, with the help of the corrupted edge node $ID_{i}$, the gateway is able to aggregate to a valid trapdoor for the message $m_{l}$ without the permission of edge node $ID_{j}$. The attack works as follows: (1) In the group public key generation phase, edge node $ID_{i}$ broadcasts $r_{i}-r_{j}$ instead of $r_{i}=x_{i}P$; (2) In the trapdoor generation phase, edge node $ID_{i}$ generates the trapdoor with $x_{i}$ as normal; (3) In the trapdoor generation phase, the gateway does not include the partial trapdoor $w_{j}$ of the edge node $ID_{j}$ in the aggregation process, i.e., it computes $T_{m_{l}}=\sum_{i\neq j}w_{i}$ which turns out to be a valid trapdoor since $r_{j}$ has been canceled out in the group generation phase.
- The cloud is considered to have a sufficient computing and storage capacity, and some tasks that are not time-sensitive and require significant computing and storage resources will be sent to the cloud for processing.

### 2.2. Design Goals

Considering the security risks posed by malicious behaviors to the resource allocation process in edge computing, our security goals must satisfy the following points.

1. *Metadata privacy*: The gateway should be able to check whether a legitimate keyword is included in the encrypted metadata without learning the content of the metadata. When the gateway has no complete trapdoor, it will be equivalent to an eavesdropper. For an eavesdropper, indistinguishability is required. That is, the eavesdropper cannot distinguish whether two encrypted keywords are the same. In other words, one of the given two keywords is randomly chosen to be encrypted, and the attacker cannot distinguish which keyword the encrypted keyword corresponds to.
2. *Full key compromise resistance*: Even if the attacker corrupts all the edge nodes in the system, the attacker still cannot decrypt the previously encrypted metadata.
3. *Semi-malicious edge node resistance:* Semi-malicious edge nodes cannot disrupt other nodes’ participation in group public key generation via an elimination attack.

### 2.3. Bilinear Pairing

Suppose that $G_{1}$ and $G_{T}$ are cyclic groups of prime order *q* and that the generator of $G_{1}$ is *P*. A pair $\hat{e}:G_{1}\times G_{1}\to G_{T}$ is a bilinear pairing if it satisfies

1. Bilinear: $\hat{e}\left(uP,vP\right)=\hat{e}{\left(P,P\right)}^{uv}$, where $u,v\in Z_{q}^{*}$.
2. Non-degenerative: For each $x\in G_{1}$, there exists exactly one $y\in G_{1}$ with $\hat{e}\left(x,y\right)=1$.

### 2.4. Identity-Based Cryptosystem

The concept of the identity-based cryptosystem (IBC) was first proposed by Shamir [34]. In IBC, the user’s public key is their identity, thus reducing the certificate management overhead in q traditional Public Key Infrastructure-based (PKI-based) cryptosystem [35]. Our scheme is designed in IBC [34], where the edge nodes use their identity as the public key, thus reducing complex problems such as certificate updates and revocations.

### 2.5. Searchable Encryption

Searchable encryption [36] is a cryptographic primitive that allows encrypted data to be searched by a user with a trapdoor without revealing information about the data. Searchable encryption mainly consists of symmetric searchable encryption (SSE) [37] and public key encryption with keyword search (PEKS) [38], which have different focuses on functionality and performance and are used to meet the requirements in different scenarios. The data owner, trapdoor generator, and decryptor in SSE must share a key, while PEKS allows anyone who knows the entity’s public key to generate the encrypted keyword. Since PEKS enables smart gateways to determine whether a keyword is present in encrypted metadata without learning its content, PEKS enables metadata privacy protection against the malicious gateway.

In an edge computing system, it is reasonable to assume that a group of edge nodes that agree on the same privacy terms will decide what information can be leaked to the gateway. This means that these edge nodes should generate a trapdoor cooperatively. However, in current searchable encryption schemes for multiple recipients [39], if an attacker obtains one of the recipients’ private keys, they can generate any trapdoor to recover the keyword. Although this problem can be solved using a thresholding scheme, it also means that the length of the ciphertext corresponding to the keyword will grow linearly with the number of receivers. Our proposed scheme achieves constant message expansion, i.e., the length of the encryption keyword does not increase with the number of edge nodes, which contributes to the scalability of the scheme.

### 2.6. Extractable Zero-Knowledge Proof

A zero-knowledge proof [40] allows the prover to prove a statement’s truth without revealing additional information to the verifier. The proofs of knowledge used in this work need to be extractable, meaning that there exists an extractor with oracle access to the prover that can output the witness of a statement. We adopt the notation proposed by Camenisch et al. [41] to represent zero-knowledge proofs of discrete logarithms. Specifically, we use $PK\{\left(x,y,z\right):h=aP+yc\wedge h^{\prime }=xP+zc\}$ to denote the zero-knowledge proofs of x,y,z that can make the equations h=aP+yc and $h^{\prime }=xP+zc$ hold. If the extractor can extract the witness without rewinding, it is called online-extractable [42], and in this paper, witnesses that need to be online-extractable will be underlined.

## 3. Proposed Scheme

### 3.1. Preliminary Definitons

In this section, the relevant definitions of the proposed scheme will be given, and our proposed scheme consists of a PPT algorithm (Init, Reg, GG, KG, ME, TA, RA), which works as follows:

- Initialization. $Init\left(1^{k}\right)\to \left(s,\Delta \right)$: The randomization algorithm Init takes the security parameter *k* as input and outputs the system master secret s and public system parameters Δ. The security parameter *k* is a parameter used to ensure the security of the scheme, usually including the secret key length and the output length of the hash function, etc.; this parameter needs to be chosen based on the tradeoffs between the desired security level and the performance requirements. The system parameters need to be public.
- Registration. $Reg\left(ID_{i}\right)\to \left(sk_{i}\right)$: The deterministic algorithm Reg is run by TA and takes as input the identity $ID_{i}$ of the edge node and outputs the private key corresponding to $ID_{i}$.
- Group Generation. $GG\left(ID_{1},ID_{2},...,ID_{k}\right)\to \left(GID,E\right)$: A group of edge nodes $ID_{1},...,ID_{k}$ with total number *k* that want to build an edge computing system can run the algorithm to generate the corresponding group identity GID and group public key *E*.
- Keyword Generation. $KG\left(ID_{1},ID_{2},...,ID_{k}\right)\to \left(Keywords\right)$: A group of edge nodes $ID_{1},...,ID_{k}$ with total number *k* generates a list of keywords for the end devices in the edge computing system.
- Message Encapsulation. ME(m,E)→(C): The algorithm can be run by any end device that knows the group public key. Inputting the group public key *E* and the metadata keyword *m*, the algorithm outputs the corresponding encryption keyword *C*. Finally, *C* and the task data need to be sent to the gateway.
- Test Authorization. $TA\left(T_{1},T_{2},...,T_{k}\right)\to \left(T\right)$: In order to authorize the gateway to test a specific keyword *m*, each edge node $ID_{i}$ in the group needs to generate the corresponding partial trapdoor $T_{i}$. The gateway aggregates these partial trapdoors to generate the final trapdoor *T*.
- Resource allocation. RA(C,T)→(0,1): The gateway runs the algorithm, and the input is the complete trapdoor *T* and the encryption keyword *C*. The algorithm outputs 1 if *C* contains the corresponding keyword *m*; otherwise, it outputs 0. Subsequently, the gateway can decide the resource allocation result based on the output and the corresponding tag of the edge node.

### 3.2. The Proposal

**Figure 2:**
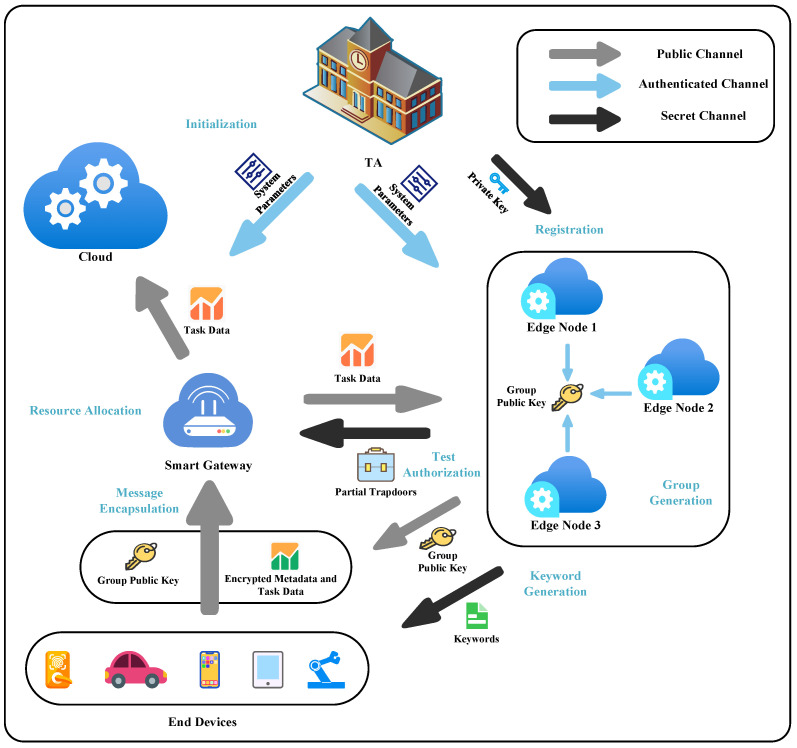
Execution process of our scheme.

As shown in Figure 2. Our scheme consists of several phases, which are described below: Initialization, Registration, Group Generation, Keyword Generation, Message Encapsulation, Test Authorization, and Resource Allocation. The specific construction is shown below:

- Initialization: Taking as input the security parameter *k*, the TA generates a bilinear pairing $\hat{e}:G_{1}\times G_{1}⟶G_{T}$, where $G_{1}$ and $G_{T}$ are both cyclic groups with prime order *q*, and *P* is the generator of $G_{1}$. TA randomly chooses $s\in Z_{q}^{*}$ as the system master secret and computes $P_{pub}=sP$; then, three cryptographic hash functions are selected, notated as $h_{1}:{\left\{0,1\right\}}^{*}⟶G_{1}$, $h_{2}:{\left\{0,1\right\}}^{*}⟶G_{1}$ and $h_{3}:G_{T}⟶{\left\{0,1\right\}}^{k}$. Then, TA generates a common reference string, which includes a description of the group $G_{1}$, denoted by CRS. Finally, the TA publishes all system parameters:
  $$\mathrm{param}=(G_{1},G_{T},\hat{e},P,P_{pub},h_{1},h_{2},h_{3},CRS).$$
- Registration: During this phase, TA should provide a registration service for edge nodes. Specifically, each edge node sends its $ID_{i}$ to TA through a secure channel, and TA first computes $q_{i}=h_{1}\left(ID_{i}\right)$ and subsequently computes $s_{i}=sq_{i}$ as the private key of edge node $ID_{i}$.
- Group Generation: Suppose an edge system consists of *k* edge nodes with IDs $ID_{1},...,ID_{k}$, and a group public key needs to be negotiated at that phase. Each edge node needs to maintain a set that contains all valid edge node subscripts, i.e., S={1,2,....,k}. Subsequently, the edge nodes perform the following operations:
  – For 1≤i≤k, the *i*-th edge node $ID_{i}$ chooses a random number $x_{i}\in Z_{q}^{*}$ and computes $r_{i}=x_{i}P$ as well as a proof $\pi_{r_{i}}=PK\left\{\left(\underline{x_{i}}\right):r_{i}=x_{i}P\right\}$. Finally, the edge node $ID_{i}$ sends $\left\{ID_{i},r_{i},\pi_{r_{i}}\right\}$ to other edge nodes via a secure channel.
  – Upon receiving a set of ${\left\{\left(r_{i},\pi_{r_{i}}\right)\right\}}_{i\in S}$ from other edge nodes, an edge node verifies the validity of $\pi_{r_{i}}$ with regard to $r_{i}$ and param for all i∈S. If $\pi_{r_{i}}$ is not valid, this also implies that the edge node is semi-malicious; therefore, the subscript of the node should be removed from the set *S*, i.e., update S:=S∖{i}. Subsequently, these edge nodes need to select a serial number (which can be instantiated based on a concatenation of the date and the value of a counter of the number of the group generation) to negotiate and publicize a unique group ID:
    (1) $$GID=ID_{1}||ID_{2}||...||ID_{i\in S}\left|\right|serial\;number.$$
    Finally, the edge node can compute and publish the group public key E=(r,Φ,S), where
    (2) $$r=\sum_{i\in S}r_{i},\Phi =\hat{e}\left(\sum_{i\in S}h_{1}\left(ID_{i}\right),P_{pub}\right).$$
- Keyword Generation: In this phase, the edge system nodes need to negotiate to generate a list of keywords for the end device. This list of keywords is sent through the secure channel to the end device that wants to use this edge system. In order to resist the keyword guessing attack [43], the edge system needs to select random strings as keywords and the end devices and edge nodes need to record the correspondence between the keywords and the required factors.
- Message Encapsulation: The end device can send task data with encrypted metadata to the gateway. In order to encrypt the keyword *m* in the metadata, an end device selects $y\in Z_{q}^{*}$ and computes the encrypted keyword (X,Y), where
  (3) $$X=yP,Y=h_{3}\left({\left(\hat{e}\left(h_{2}\left(GID,m\right),r\right)\cdot \Phi \right)}^{y}\right).$$
- Test Authorization: Suppose the set *S* contains *k* edge nodes, $GID=ID_{1}||ID_{2}||...||ID_{k}\left|\right|serial\;number$. To authorize the gateway to test the keyword list $m_{1},...,m_{n}$, the *i*-th edge node needs to compute the corresponding partial trapdoor $w_{i}=s_{i}+x_{i}h_{2}\left(GID,m_{l}\right)$ for the keyword $m_{l}$, where l∈{1,2....,n}. Subsequently, the edge node sends the corresponding $\left(w_{i},tag_{i}\right)$ to the gateway via a secure channel in a specific keyword order, where tag∈{0,1}. The tag=1 or tag=0 represents whether the i-th edge node is willing to perform the task represented by *m* or not, respectively. Receiving ${\left\{w_{i},tag_{i}\right\}}_{1\leq i\leq k}$, the gateway computes
  (4) $$T_{m_{l}}=\sum_{i=1}^{k}w_{i}.$$
  $T_{m_{l}}$ is the trapdoor used to determine whether the encrypted metadata contain the keyword $m_{l}$.
- Resource Allocation: After receiving the encryption keyword $\left(X_{j},Y_{j}\right)$ in the encrypted metadata, the gateway needs to determine whether the equation
  (5) $$h_{3}\left(\hat{e}\left(T_{m_{l}},X_{j}\right)\right)\overset{?}{=}Y_{j}$$
  holds. If the equation holds, the encrypted metadata contain the keyword $m_{l}$; otherwise, the encrypted metadata do not contain $m_{l}$. After testing all the keywords, the gateway can complete the resource allocation process according to the tag corresponding to the trapdoor. If no edge node is available to execute the task, it will be sent to the cloud for execution.

## 4. Security Analysis

This section starts with the definition of the security model for the proposed scheme. The security model captures the properties of user privacy, corrupted edge nodes, and full key compromise resistance. Following this, we provide a formal proof that our proposed scheme satisfies the required security notion.

### 4.1. Security Model

In our security model, an adversary can be either a gateway or a eavesdropper. We call a gateway adversary a Type I adversary and an eavesdropper adversary a Type II adversary. We define the security of our proposed scheme through the following two games.

Game 1 plays between a challenger C and a Type I adversary A, which captures the behavior of the Type I adversary. In Game 1, the adversary A will try to learn the contents of the encrypted metadata. Game 1 consists of three phases.

*Initial*: C run Initialization to obtain *master-secret* and the system parameter list. It then sends the system parameters to A while keeping *master-secret* secret.

*Training*: Allow A to adaptively execute a polynomially bounded number of the following types of queries:

- Private key queries: This query is used to model our proposed scheme’s full key compromise resistance property. Specifically, A is allowed to perform this query to obtain the private key of the edge node with the identity $ID_{i}$, and the query’s output is the corresponding node’s private key.
- Group public key queries: A can use this query to obtain the group public key for a group of edge nodes. C responds with the corresponding group public key.
- Trapdoor queries: A can request the trapdoor corresponding to metadata *m*, and, as a response, C runs the Test Authorization algorithm to obtain and return the trapdoor.

*Output*: A selects a group ID $GID^{*}$ and sends that group to challenger *C*. *C* chooses a keyword $m^{*}$, encrypts it, generates a trapdoor $T_{m^{*}}$, and sends $T_{m^{*}}$ to the adversary; if A is able to correctly decrypt $T_{m^{*}}$, we say that A has won game 1.

Define the advantage of A winning the above game as

(6) $$Adv\left(A\right)=|\mathrm{Pr}\left[\xi^{\prime }=\xi \right]-\frac{1}{2}|.$$

If for any probabilistic polynomial time adversary A, Adv(A) can be negligible, we say that our scheme is secure against Type I adversaries.

Game 2 plays between a challenger C and a Type II adversary A, which captures the behavior of the Type II adversary. In Game 2, two encrypted keywords are given to the adversary A, and A is required to distinguish whether the two contain the same keyword. Game 2 consists of three phases.

*Initial*: This phase is the same as in Game 1.

*Training*: Allow A to adaptively execute a polynomially bounded number of the following types of queries:

- Private key queries: This query is used to model our proposed scheme’s full key compromise resistance property. Specifically, A is allowed to perform this query to obtain the private key of the edge node with identity $ID_{i}$, and the query’s output is the corresponding node’s private key.
- Group public key queries: A can use this query to obtain the group public key for a group of edge nodes. C responds with the corresponding group public key.
- Trapdoor queries: A can request the trapdoor corresponding to metadata *m*, and, as a response, C runs the Test Authorization algorithm to obtain and return the trapdoor.
- Semi-malicious corruptions queries: C needs to maintain an initially empty list $L_{m}$ with records of the format $\left(ID_{i},r_{i},\pi_{r_{i}}\right)$. A is allowed to add malicious records to $L_{m}$.

*Output*: The adversary A selects a group ID $GID^{*}$ and two keywords $m_{0},m_{1}$. Next, $\left(GID,m_{0},m_{1}\right)$ is sent to C. We require that A cannot query the trapdoor corresponding to $m_{0}$ and $m_{1}$ under $GID^{*}$. However, A can corrupt all the edge nodes related to $GID^{*}$. Then, C can randomly select the message $m_{\xi }$, where ξ∈{0,1}, and encrypt that message using the group public key corresponding to $GID^{*}$ to get the encryption keyword $C^{*}$, which will be sent to A as a response. Finally, A returns its guess: $\xi^{\prime }\in \left\{0,1\right\}$.

If for any probabilistic polynomial time adversary A,

(7) $$Adv\left(A\right)=|\mathrm{Pr}\left[\xi^{\prime }=\xi \right]-\frac{1}{2}|$$

can be negligible, our scheme is secure against Type II adversaries.

### 4.2. Security Proofs

The security of our proposed scheme is based on the Bilinear Diffie–Hellman (BDH) assumption, which is defined as follows.

**BDH Problem**: It is difficult to compute $\hat{e}{\left(P,P\right)}^{\alpha \beta \gamma }$ when given P,αP,βP,γP, where $\alpha ,\beta ,\gamma \in Z_{q}$.

**BDH Assumption**: Suppose B is an algorithm that can solve the BDH problem with the advantage

$$Adv\left(B\right)=\mathrm{Pr}\left[B\left(P,\alpha P,\beta P,\gamma P\right)=\hat{e}{\left(P,P\right)}^{\alpha \beta \gamma }\right].$$

Then, for any polynomial time algorithm B, Adv(B) can be negligible.

**Theorem** **1.** *In time τ, assuming that A can win Game 1 with an advantage denoted as Adv(A), this means that there exists an algorithm that breaks the one-way property of the hash function.*

**Proof.**  Next, we will prove that if an adversary A can break the user privacy, then it means that C can utilize A to break the one-way property of the hash function. In the real world, this is considered difficult.

*Initial*: C runs Initialization to get *master-secret* and the system parameter list, then sends the system parameters to A while keeping *master-secret* secret.

*Training*: Type I adversaries A are allowed to do the various query requests defined above, and C responds accordingly as defined in Section 3.2.

*Output*: A selects a group ID $GID^{*}$, generates the corresponding group encryption public key $E^{*}=\left(r^{*},\Phi^{*}\right)$, and sends $\left(GID^{*},E^{*}\right)$ to C. C randomly chooses a keyword $m^{*}$ and randomly chooses $o^{*}\in G_{1}$ as the trapdoor corresponding to $m^{*}$. If C outputs its guess as ${m^{*}}^{\prime }=m^{*}$, an algorithm exists that breaks the one-way property of the hash function. □

**Theorem** **2.** *Suppose the group size is N; in time τ, the Type II adversary A requested $q_{h_{3}}$ queries for $h_{3}$ and requested $q_{t}$ trapdoor queries. Suppose that the adversary A can win Game 2 with an advantage denoted by Adv(A). This then means that there exists an algorithm to solve the BDH problem, which has an advantage of*

$$\frac{1}{q_{h_{3}}Ne^{2}}{\left(\frac{2}{2q_{t}+2}\right)}^{2}Adv\left(A\right).$$

**Proof.**  Next, we will prove that if the adversary A can break our scheme, then C can utilize A to solve the BDH problem. In the real world, this is considered difficult.

*Initial*: C sets $P_{pub}=\alpha P$, then generates the system parameters

$$params=(G_{1},G_{T},\hat{e},P,P_{pub},h_{1},h_{2},h_{3}),$$

and sends the system parameters to A. Assume that the number of edge nodes in the system is N. The *i*-th edge node $ID_{i}$ performs the registration when C flips a coin $coin_{i}$; the probability of producing a 1 is δ, and the probability of producing a 0 is 1−δ, where 1≤i≤N.

*Training*: We consider $h_{1},h_{2}$, and $h_{3}$ to be random oracles, and C responds to the request of A as follows:

- $h_{1}$ queries: C needs to maintain an initially empty list $L_{h_{1}}$. When $ID_{i}$ is input, C first checks whether the record $\left(ID_{i},d_{i},q_{i},s_{i}\right)$ exists in $L_{h_{1}}$, and, if it does, it returns $q_{i}$ to A; otherwise, C randomly selects $d_{i}\in Z_{q}^{*}$, computes $q_{i}=d_{i}P,s_{i}=d_{i}P_{pub}$, adds the record $\left(ID_{i},d_{i},q_{i},s_{i}\right)$ to $L_{h_{1}}$, and returns $q_{i}$ to A.
- $h_{2}$ queries: C needs to maintain an initially empty list $L_{h_{2}}$. When $ID_{i}$ is input, C first checks whether the record $\left(GID_{i},m_{i},w_{i},o_{i},coin_{i}^{h_{2}}\right)$ exists in $L_{h_{2}}$, and, if it does, it returns $o_{i}$ to A. Otherwise, C flips a coin $coin_{i}^{h_{2}}$, assuming that the probability of the coin yielding 1 is δ and the probability of yielding 0 is 1−δ, and, subsequently, C randomly selects $w_{i}\in Z_{q}^{*}$.
  – If $coin_{i}^{h_{2}}=1$, compute $o_{i}=\left(w_{i}+\beta \right)P$, add record $\left(GID_{i},m_{i},w_{i},o_{i},coin_{i}^{h_{2}}\right)$ to $L_{h_{2}}$, and return $o_{i}$ as response.
  – Else, compute $o_{i}=w_{i}P$, add record $\left(GID_{i},m_{i},w_{i},o_{i},coin_{i}^{h_{2}}\right)$ to $L_{h_{2}}$, and return $o_{i}$ as response.
- $h_{3}$ queries: C needs to maintain an initially empty list $L_{h_{3}}$. When $\Lambda_{i}$ is input, C first checks whether record $\left(\Lambda_{i},Y_{i}\right)$ exists in $L_{h_{3}}$, and, if it does, returns $Y_{i}$ to A; otherwise, C randomly selects $Y_{i}\in {\left\{0,1\right\}}^{*}$, adds the record $\left(\Lambda_{i},Y_{i}\right)$ to $L_{h_{3}}$, and returns $Y_{i}$ to A.
- Private key queries: The query takes $ID_{i}$ as input, and, upon receiving this query, C performs an $h_{1}$ query with $ID_{i}$ as input, and then recovers the corresponding $\left(ID_{i},d_{i},q_{i},s_{i}\right)$ from $L_{h_{1}}$, returning $s_{i}$ as a response.
- Semi-malicious corruptions queries: A inputs $\left(ID_{i},r_{i},\pi_{r_{i}}\right)$, and C adds the record $\left(ID_{i},r_{i},\pi_{r_{i}}\right)$ to $L_{m}$.
- Group public key queries: C maintains a list $L_{G}$ with records of the form
  $$(GID_{l},r_{1},r_{2},...,r_{k},x_{1},x_{2},...,x_{k},E,\Phi ).$$
  The query receives $GID_{j}=(ID_{1}||ID_{2}||...||ID_{k}\left||serial\;number\right)$ as input; for 1≤i≤k, C first determines whether there is a record associated with $ID_{i}$ in $L_{m}$, and, if not, C randomly chooses $x_{i}\in Z_{q}^{*}$, otherwise recovering the corresponding record $\left(ID_{i},r_{i},\pi_{r_{i}}\right)$ from $L_{m}$, and we note that, in this work, we use extractable knowledge proofs so that we can extract the $x_{i}$ from $\pi_{r_{i}}$. Then, if $coin_{i}=0$, C computes $r_{i}=x_{i}P$, and if $coin_{i}=1$, C calculates $r_{i}=\left(x_{i}-\alpha \right)P$. Finally, C computes
  (8) $$E=(r=\sum_{i=1}^{k}r_{i},\Phi =\hat{e}\left(\sum_{i=1}^{k}h_{1}\left(ID_{i}\right),P_{pub}\right)).$$
  Add the record
  $$\left(GID_{j},r_{1},r_{2},...,r_{k},x_{1},x_{2},...,x_{k},E,\Phi \right)$$
  to $G^{lits}$.
- Trapdoor queries: C maintains a list $L_{t}$ with records of the form $\left(GID_{i},m_{i},T_{i}\right)$. The query receives $\left(GID_{j},m_{j}\right)$ as input, C first recovering
  $$\left(GID_{j},r_{1},r_{2},...,r_{k},x_{1},x_{2},...,x_{k},E,\Phi \right)$$
  from $L_{G}$. For 1≤i≤k, C recovers the $ID_{i}$ corresponding to the records $\left(ID_{i},d_{i},q_{i},s_{i}\right)$ from $L_{h_{1}}$, and the $\left(GID_{j},m_{j}\right)$ corresponding records $\left(GID_{j},m_{j},w_{j},o_{j},coin_{j}^{h_{2}}\right)$ from $L_{h_{2}}$. Then, C determines whether a record $\left(GID_{j},m_{j},T_{j}\right)$ corresponding to $\left(GID_{j},m_{j}\right)$ exists in $L_{t}$, and, if it does, C returns $T_{j}$ as a response. If 1≤i≤k, then C executes the following steps:
  – If $coin_{i}=0$, since C has knowledge of $x_{i}$ and the private key of $ID_{i}$, C can use the *Trapdoor* algorithm to generate $w_{i}$.
  – Else, if $coin_{i}^{h_{2}}=0$, computer $w_{i}=s_{i}+w_{j}r_{i}$.
  – Else, abort. We denote the event by Event 1.
  If Event 1 does not occur, C computes $T_{j}=\sum_{i=1}^{k}w_{i}$ and adds the record $\left(GID_{j},m_{j},T_{j}\right)$ to $L_{t}$.
- *Challenge*: A chooses a group ID $GID^{*}$ corresponding to $L_{ID}^{*}=\left\{ID_{1}^{*},ID_{2}^{*}...,ID_{x}^{*}\right\}$, two keywords $m_{0}^{*},m_{1}^{*}$, and the group public key $E^{*}=\left(r^{*},\Phi^{*}\right)$. Then, A sends $\left(GID^{*},E^{*},m_{0}^{*},m_{1}^{*}\right)$ to C. C randomly selects ξ∈{0,1}, $Y^{*}\in {\left\{0,1\right\}}^{l}$, sends $\left(\gamma P,Y^{*}\right)$ as a response to A, and finally, A outputs its guess $\xi^{\prime }$.
- *Output*: If $\xi^{\prime }=\xi$, C recovers the records $\left(GID^{*},r_{1}^{*},...,r_{k}^{*},x_{1}^{*},...,x_{k}^{*},E^{*},\Phi^{*}\right)$ from $L_{G}$, for 1≤l≤k, C recovers the record $\left(ID_{l},\mu_{l},f_{l},s_{l}\right)$ from $L_{h_{1}}$. For $\left(GID^{*},m_{\xi }^{*}\right)$, C recovers the corresponding records $\left(GID^{*},m_{\xi }^{*},w_{\xi }^{*},o_{\xi }^{*},H_{2}coin_{\xi }^{*}\right)$ from $L_{h_{2}}$. For 1≤l≤k, we denote the value of the coin flip corresponding to $ID_{l}$ as $coin_{l}^{*}$. It requires that only one coin corresponds to a value of 1. Then, C randomly selects the pair $\left(\Lambda_{i},Y_{i}\right)$ from $L_{h_{3}}$. Finally, C outputs
  (9) $$\Lambda_{i}/\left(e\left(\left(\gamma \sum_{i=1}^{k}d_{i}^{*}\right)P,P_{pub}\right)e\left(w_{\xi }^{*}r,\gamma P\right)e\left(\left(\beta \sum_{i=1}^{k}x_{i}^{*}\right)P,\gamma P\right)\right)$$
  as the answer to the BDH problem.
  When Event 1 does not happen, A will not notice the difference between the simulation and the real world, so we have
  (10) $$Pr\left[\xi^{\prime }=\xi \right]\geq Adv\left(A\right),Pr\left[\neg Event1\right]\geq {\left(\left(1-\delta \right)\left(1-N\delta \right)\right)}^{q_{t}}.$$
  We note that for C to output a solution to the BDH problem, it is required that, for an index l∈[1,k], $coin_{l}^{*}=1$ and ${coin_{\xi }^{h_{2}}}^{*}=1$. And these occur with a probability of at least $N\delta^{2}$. Thus, we have C outputting a solution to the BDH problem with the probability
  (11) $$\frac{1}{q_{h_{3}}}N\delta^{2}{\left(\left(1-\delta \right)\left(1-N\delta \right)\right)}^{q_{t}}Adv\left(A\right)\geq \frac{1}{q_{h_{3}}Ne^{2}}{\left(\frac{2}{2q_{t}+2}\right)}^{2}Adv\left(A\right).$$

□

## 5. Security Comparison and Performance Evaluation

Although the work of Barman et al. [12] and Jiang et al. [13] is robust, as mentioned before, it is unrealistic to introduce these complex schemes in edge computing systems with limited computational and bandwidth resources. Therefore, we compare the security of our proposed scheme with Zhang et al.’s proposed scheme [14], which also uses searchable encryption. We have compared the security of the two schemes via formal security proofs, and the comparison results are shown in Table 1. The results show that our scheme can resist semi-malicious edge nodes, which Zhang et al.’s scheme cannot do. Our proposed scheme and Zhang et al.’s proposed scheme have similar computational overheads in the other phases, with the main gap being the extractable zero-knowledge proofs introduced in the group generation phase. To evaluate these extra computational overheads, we performed experiments on the zero-knowledge proof scheme proposed by Cramer et al. [44] The experiment was performed using the Miracl library on a PC equipped with an i5-9400F CPU and 16 GB RAM. The experimental results show that the proof generation time is 2.98 s, while the verification time is 26.32 ms. Since the group generation phase is executed only once, such extra computational overhead is acceptable. In recent years, some more efficient zero-knowledge proof schemes have been proposed [45]. In practice, the appropriate zero-knowledge proof schemes can be selected as the building blocks of our proposed scheme as needed.

Next, we evaluate our proposed scheme in terms of performance. To our knowledge, no metadata privacy-preserving resource allocation scheme exists for resistance to semi-malicious edge nodes. Therefore, we only evaluate the performance of our proposed scheme. In addition, since the other phases need to be executed only once, we only evaluate the efficiency of the Message Encapsulation phase, the Test Authorization phase, and the Resource Allocation phase. Specifically, in this section, we use the petrelic library to evaluate the computational overhead of our proposed scheme. We performed the measurements on a PC with an Intel i5-9400F CPU, 16GB of RAM, and Ubuntu 18.04.

**Figure 3:**
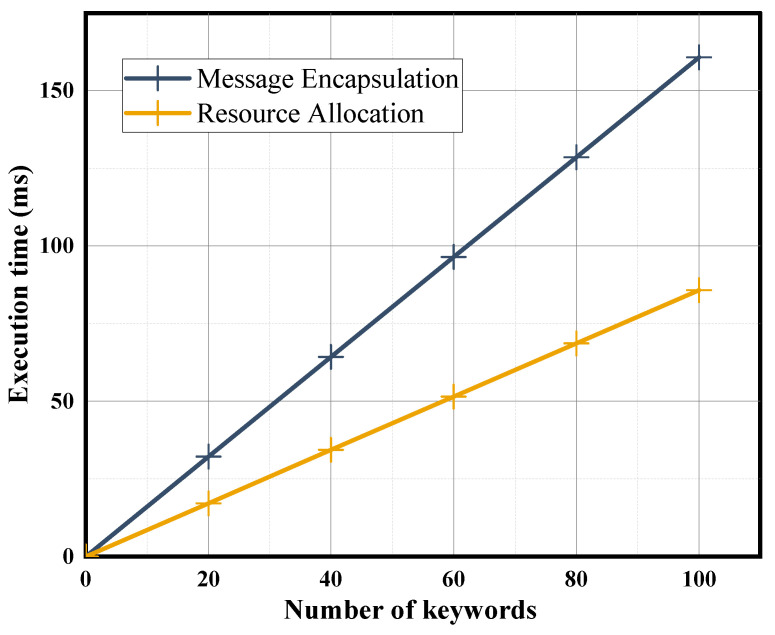
Execution time of Message Encapsulation phase and Resource Allocation phase on the PC.

**Figure 4:**
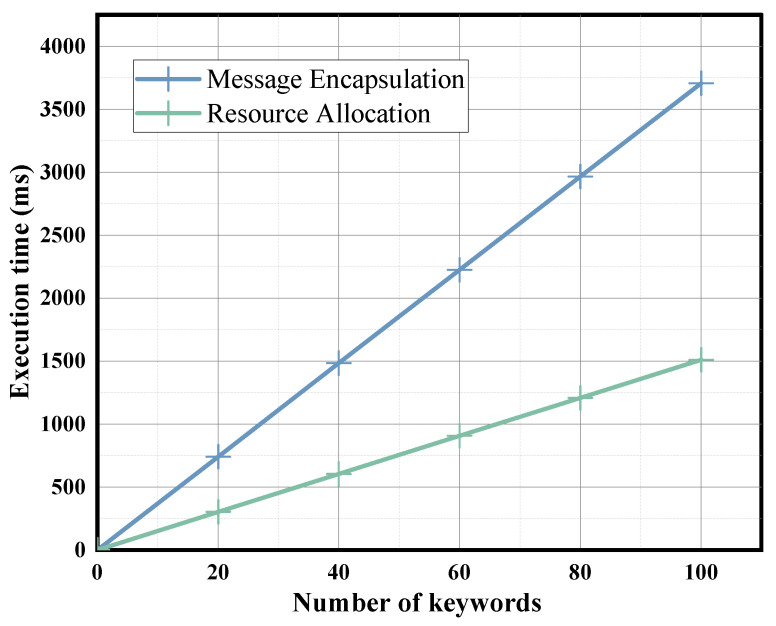
Execution time of Message Encapsulation phase and Resource Allocation phase on the Raspberry Pi.

We increase the number of encrypted keywords from 1 to 100 and calculate the required execution time in order to evaluate the performance of the Message Encapsulation phase. The results are shown in Figure 3. Our proposed scheme requires an execution time of about 1.607 ms for a single keyword. Similarly, we evaluate the Resource Allocation phase, where the execution time is about 0.858 ms for a single keyword. Since the metadata attached to the edge computation will not contain too many keywords, the corresponding two phase computation overhead will not be particularly large. In addition, to evaluate the scheme’s performance on edge devices with limited computational resources, we performed experiments on a Raspberry Pi 3 Model B+ using the PBC library. The results are shown in Figure 4. The execution times of our scheme in the Message Encapsulation phase and Resource Allocation for a single keyword are 37.071 ms and 15.102 ms, respectively. Since the metadata attached to the edge computation will not contain too many keywords, the corresponding two-phase computation overhead is acceptable for edge devices.

**Figure 5:**
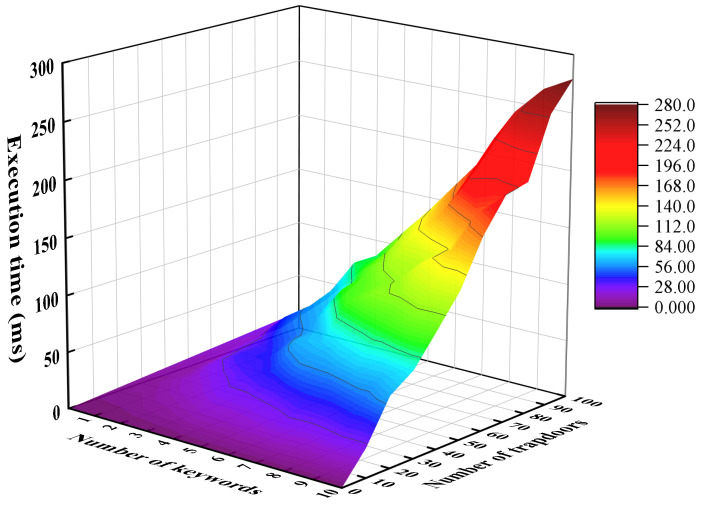
Execution time of Test Authorization phase on the PC.

**Figure 6:**
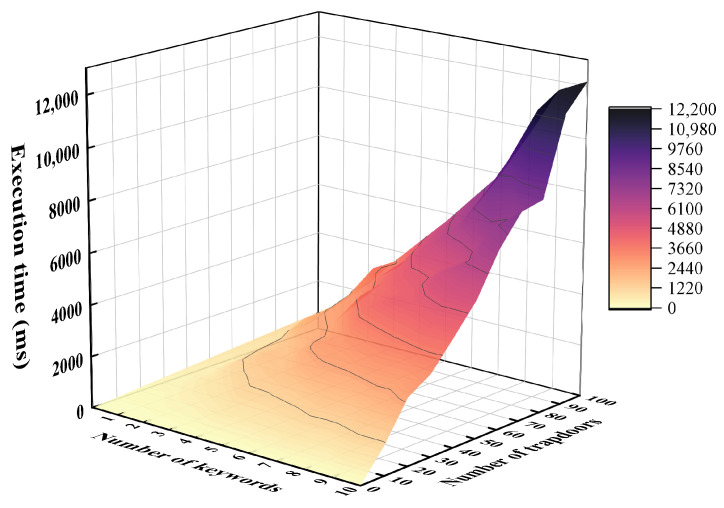
Execution time of Test Authorization phase on the Raspberry Pi.

Since the number of keywords contained in the metadata and the number of edge nodes in the edge computing system will not be too many, we set the number of encrypted keywords to grow from 1 to 10, and the number of partial trapdoors to increase from 1 to 100, to evaluate the performance efficiency of the Test Authorization phase. Similarly, the experiment was performed on a PC and a Raspberry Pi, and the results are shown in Figure 5 and Figure 6, respectively. We can see that the performance efficiency of the Test Authorization phase is usually sufficient to meet the requirements.

## 6. Conclusions

Resource allocation is the core problem of edge computing, but existing resource allocation schemes in edge computing often lack the protection of metadata privacy and consideration of semi-malicious edge nodes. In this paper, we propose a metadata-privacy resource allocation scheme based on searchable encryption and use a zero-knowledge proof to resist semi-malicious edge nodes. Our proposed scheme achieves constant message expansion, which contributes to scalability in practice. Through a formal security analysis, we demonstrate that the scheme satisfies the necessary security and privacy requirements. We show that the scheme’s efficiency can meet the requirements through experiments on PC and Raspberry Pi. Overall, our proposed scheme provides a practical and robust solution for resource allocation in edge computing.

In future work, we will consider introducing trust mechanisms and integrating them with existing edge computing frameworks, which will contribute to the dynamism and scalability of the scheme. In addition, introducing more powerful malicious edge nodes and de-trusting TA are two interesting issues that will provide more robust security for edge computing systems.

## Figures and Tables

**Table 1:** Comparison of security.

	Metadata Privacy	Full Key Compromise Resistance	Semi-Malicious Edge Nodes Resistance
Zhang et al.’s scheme [14]	✓	✓	✗
Our proposed scheme	✓	✓	✓

## Data Availability

Data are contained within the article.
